# The Psychological Impact of Smog Pollution on Investors

**DOI:** 10.3389/fpsyg.2022.803336

**Published:** 2022-02-04

**Authors:** Bin Li, Yang Liu, Tingyu Zhang, Yijing Wang

**Affiliations:** ^1^School of Economics and Management, Beijing University of Chemical Technology, Beijing, China; ^2^School of Government, Peking University, Beijing, China; ^3^School of Economics and Management, Beijing University of Chemical Technology, Beijing, China; ^4^Planning and Finance Department, Xiangtan University, Xiangtan, China

**Keywords:** smog pollution, investor sentiment, media tone, PM 2.5, moderating effect

## Abstract

This research empirically analyzes the psychological impact of smog pollution on investors. Results indicate that smog pollution has negative impact on investor sentiment which is weakened by the positive tone in media reporting. Empirical evidence for the impact of smog pollution on investor sentiment and the related moderating role of media tone is presented in this study.

## Introduction

Smog pollution not only affects people’s physical health (Chen et al., 2016), as well as psychological health and their behavior (Levy and Yagil, 2011). Some related studies found that air quality can change investor sentiment and behavior, thus affecting trade activities and the stock market (Li and Peng, 2016). However, extant research on the relationships between air quality and investor sentiment is more focused on financial markets. It is lack of research focusing on the impact of smog pollution on investor sentiment at the level of individual share. This study uses panel data of China’s listed companies to test the psychological impact of smog pollution on investors, as well as analyzes the moderating effects of media tone in related news reporting.

The original contributions of this study include three aspects. First, instead of previous studies that focus on changes in investor sentiment as climatic factors that influence financial markets, or studies that use air quality as an indirect proxy variable for investor sentiment, this study focuses on the influence of smog pollution on investor emotion on the fluctuate of stock markets. Second, while prior studies prove that markets react to pollution events (Wang et al., 2019), this study confirms that smog pollution, a type of change in external natural environment, has significant negative impact on investor sentiment. Third, this study confirms the moderating effect of positive intonation from media exists on the correlation between smog pollution and investor sentiment, as well as provides empirical evidence for a comprehensive assessment of the elements of smog influence and the heterogeneity of reporting tones in mass media.

## Literature Review and Hypothesis

### Investor Sentiment

To define investor sentiment, some studies used stock market mispricing (Polk and Sapienza, 2009) and the false expectations of asset fundamentals (Baker and Stein, 2004). To measure market investor sentiment, objective and subjective indicators have been used. For instance, objective indicators include closed-end fund discounts, IPO circulation, and trading volume (Ljungqvist et al., 2006), while subjective indicators include the investor intelligence index (Brown and Cliff, 2004), the analyst sentiment index (Wurgler and Baker, 2006), and the consumer confidence index (Wang et al., 2021). In a related study, Obaid and Pukthuanthong (2021) measured investor sentiment with machine learning and photos from the news.

In the external environment context of our fucus topic, some scholars suggested macroeconomic development expectations such as macroeconomic conditions and monetary policy expectations affected investor sentiment (Kurov, 2010). Another factors that affects investor sentiment is media attention (Peress and Fang, 2009). Moreover, at least one study found that an overconfident Fed chair is significantly associated with higher investor sentiment (Bennani, 2020). In addition, other studies analyzed how the coronavirus pandemic affected investor sentiment (Sun et al., 2021).

### Smog Pollution and Investor Sentiment

Air pollution increases the negative sentiments among investors, thus triggering more conservative investment-related activities (Levy and Yagil, 2011; Li and Peng, 2016; Peng et al., 2021). This study proposes that smog pollution can change the expectations and judgments of investors by influencing the development of companies and investors’ perceptions. These, in turn, affects investor sentiments in various financial, trade, and industry sectors.

Firstly, smog pollution affects the development of companies. For instance, some studies found that smog pollution affects the debt financing ability (Li et al., 2019a), obtaining of subsidies (Li et al., 2019b), cash holdings (Li et al., 2021), and the market value of firms (Li et al., 2018; Peng et al., 2021). In addition, regulations of smog pollution governance internalizes the costs of air pollution and, as a result, company profits decline sharply. Following the concept of stock pricing and price signals, external news releasing environmental degradation and the implementation of environmental governance policies lead to the negative expectations of investors on the company development, the whole if which restrain the investment activities.

Secondly, information disclosed about smog pollution affects investors’ perceptions. According to the attentional resources theory, investor attention tends to be limited. For instance, investors may not fully respond to all market information due to intervening variables (Engelberg et al., 2012) such as concerns about environmental protection and smog pollution that usually interfere with investor attention on other sensitive market information. This distraction will affect investment decisions (Hirshleifer, 2001) due to negative expectations and underestimation of listed company values in air-polluted regions. Thus, the first hypothesis of this study is posed as follows.

**H1: Smog pollution in the areas that companies located in is negatively influent investor sentiment.**

### Moderating Effect of Media Tone

For many small and medium investors in China’s capital market, media concerning is important channels for obtaining market information. For instance, media reports reveal a company’s operating status, improving the efficiency of company information distribution and dissemination, and help investors access “soft information” to capture a company’s fundamentals (Tetlock et al., 2008). When corporate information is reported to the public with a positive media tone form media, investors are inclined to develop positive expectations on a company’s future, inhibiting negative mood caused by smog pollution. Thus, the second hypothesis is expressed as follows.

**H2: Media tone has inhibitory effects on the negative relationships between smog pollution and investor sentiment.**

## Methodology

### Model Design

Base on the related literature (Kurov, 2010; Sun et al., 2021), this study uses regression models to test the effect of smog pollution on investor sentiment and the moderating effects of media tone. Model (1) is used to test the relationships between smog pollution and investor sentiment; and α_1_ is expected to be significantly negative, supporting Hypothesis 1. In Model (2), the intersection items of smog pollution and media tone (*PM_2.5i,t–1_ × TONES_*i,t–1*_*) is added to test the moderating effects of media tone on the relationships between smog pollution and investor sentiment. β_1_ is expected to be significantly negative and β_3_ is expected to be significantly positive, thus verifying Hypothesis 2.


(1) $$I\,S_{i,t}=\alpha_{0}+\alpha_{1}\,P\,M\,2.5_{i,t-1}+\alpha_{2}\,C\,o\,n\,t\,r\,o\,l\,s+\sum Y\,e\,a\,r+\sum I\,n\,d\,u\,s\,t\,r\,y+\varepsilon$$

(2) $$I\,S_{i,t}=\beta_{0}+\beta_{1}\,P\,M\,2.5_{i,t-1}+\beta_{2}\,T\,O\,N\,E\,S_{i,t-1}+\beta_{3}\,P\,M\,2.5_{i,t-1}\times T\,O\,N\,E\,S_{i,t-1}+\beta_{4}\,C\,o\,n\,t\,r\,o\,l\,s+\sum Y\,e\,a\,r+\sum I\,n\,d\,u\,s\,t\,r\,y+\varepsilon$$

### Variables

The dependent variable is investor sentiment (*IS*_i,t_). Following Goyal and Yamada (2004) and Rhodes-Kropf et al. (2005), this study separates the market valuation level (Tobin’s Q) of a company into its inherent values of growth and market mispricing, and then regresses Tobin’s Q on the variable group that describes company fundamentals such as return on assets, asset-liability ratio, and main business income growth rate, and fix effect of industry and year. Using the fitted value, which is calculated according to three mentioned indicators of return on assets, asset-liability ratio, and main business income growth rate as the benchmark Q (Q_f_), the residual, Q_r_ = Q–Q_f_, is used to measure mispricing due to investor sentiment (*IS*).

The independent variable is smog pollution (*PM*_2.5i,t–1_), which is based on the air quality monitoring data released by the China National Environmental Monitoring Centre (CNEMC). Considering the hysteretic effect of air pollution on the human body, this paper uses the first-order lag term of PM_2.5_ as the independent variable.

Because of the lag in media news reports, media tone (*TONES*) is delayed by one period. The measure of media tone is derived from the overall sentiment score indicator in the news and the social media quantitative public opinion database. Using text analysis technology, the media tone indicator is scored after analyzing the news published by 327 important newspapers. To provide continuous and comparable sentiment scores for media reports, text with a positive tone are assigned positive scores while negative scores are assigned to texts with a negative tone. Finally, the aggregated sentiment scores of media report items are matched with the most relevant listed companies mentioned in the reports. The daily data of the news sentiment tendency score in the database is summarized by company, and the media tone annual index is obtained.

From related studies (Lewellen, 2011; Bennani, 2020; Sun et al., 2021), a series of control variables are selected to reduce the effects of other factors. For instance, when investing in company stock, investors may consider fundamental corporate information, such as enterprise ownership (*SOE*), enterprise scale (*SIZE*), years of establishment (*AGE*); company profitability and management activities, including profit margin on net assets (*ROE*), asset-liability ratio (*LEV*), future development opportunities (*GROW*), operating net cash flow (*CRAA*), capital investment (*INV*), management expense ratio (*ADM*), audit costs (*DCOST*), and proportion of independent directors (*IDRATIO*). In this scenario, major shareholders, institutional investors, and management staff can have information advantages outside and inside the enterprise. Thus, their shareholding ratio may affect the judgment of investors. Understandably, control variables also include the shareholding ratio of the largest shareholder (*SHRCRL*), the institutional shareholding ratio (*INHOLDS*), and the executive shareholding ratio (*MAHARE*). Moreover, the characteristics and performance of company stocks can also affect investor sentiment. Thus, four typical control variables and included: earnings per share (*EPS*), book value per share (*BPS*), book to market value (*BTM*), and stock liquidity (*TRADE*). In addition, the control variables include year and industry dummy variables. Table 1 defines all the variables.

**TABLE 1:** Definition of variables.

Variables	Descriptions	Definitions
*IS*	Investor sentiment	Decompose Tobin’s Q, take the residual
*PM*_2.5_	Smog pollution	Annual mean value of PM_2.5_ monthly concentration monitoring data for company’s location
*TONES*	Media tone	Annual mean value of news overall sentiment orientation score
*SOE*	Company ownership	Equal to 1 if the company is state-owned, 0 otherwise
*SIZE*	Company scale	The logarithm of total assets of company at the end of year t
*AGE*	Age of corporate	Year of establishment
*ROE*	Rate of Return on Common Stockholders’ Equity	Profit margin on net assets
*LEV*	Asset-liability ratio	Total liabilities at the end of year t/total assets at the end of year t
*GROW*	Future development opportunities	Operating income growth rate of company in year t
*CRAA*	Operating net cash flow	Operating net cash flow of company at the end of year t
*INV*	Capital investment	Cash for the purchase and construction of fixed assets, intangible assets and other long-term assets at the end of year t/total assets at the end of year t-1
*ADM*	Management expense ratio	Management expense ratio at the end of year t/main business income at the end of year t
*DCOST*	Audit cost	Audit fees company paid in year t, in RMB 10 thousand yuan
*IDRATIO*	Proportion of independent directors	Number of independent directors at the end of year t/number of board members at the end of year t
*SHRCRL*	Ownership concentration	Shareholding ratio of the largest shareholder of company at the end of year t
*INHOLDS*	Institutional shareholding ratio	Institutions holding shares at the end of year t/total number of shares at the end of year t
*MAHARE*	Executive shareholding ratio	Executives holding shares at the end of year t/total number of shares at the end of year t
*EPS*	Earnings per Share	Net profit at the end of year t/number of ordinary shares at the end of year t
*BPS*	Book value per share	Stockholders’ equity at the end of year t/number of ordinary shares at the end of year t
*BTM*	Book to market value	The ratio of book value to the market value of the company at the end of year t
*TRADE*	Stock liquidity	Average monthly trading volume in year t/the total number of outstanding shares at the end of year t

### Data

The financial data for all listed companies in this study is obtained from the China Stock Markets and Accounting Research (CSMAR) database, while the urban smog concentration monitoring data of 74 key cities in China from 2013 to 2017 is obtained from the CNEMC. In this dataset, the data between 2013 and 2017 have higher internal consistency because the Science and Technology Innovation Board was established in China in 2018, The data on news reporting tone is derived from the News and Social Media Quantitative Public Opinion Data published by the Chinese University of Hong Kong. To calculate relevant variables, we exclude companies with missing data and companies in the financial industry, as well as companies located in cities that do not disclose smog monitoring data, and. The final sample contains 5704 observations.

Table 2 provides descriptive statistics for the research sample. For instance, the average value of investor sentiment (*IS*) is -0.192, indicating that investors have a negative view of a company. The average value of *PM*_2.5_ is 52.21 g/μ^3^, which is on the second level for air quality (35–75 g/μ^3^) as stipulated in China, while the maximum value of 130 g/μ^3^ is on the fourth level (115–150 g/μ^3^), which indicates moderate smog pollution. The average value of media tone (*TONES*) is 23.61, indicating that the media’s news reporting tone of all the companies in the sample is generally positive.

**TABLE 2:** Descriptive statistics.

Variable	N	Mean	Std	Min	Median	Max
*IS*	5,704	–0.192	3.192	–4.285	–0.771	71.282
*PM*_2.5_	5,704	52.210	17.934	20.080	51.810	130.000
*TONES*	5,704	23.610	94.403	–1100.000	5.966	3440.000
*SOE*	5,704	0.340	0.474	0.000	0.000	1.000
*SIZE*	5,704	22.320	1.279	17.760	22.180	28.071
*AGE*	5,704	16.840	5.467	2.000	17.000	37.000
*ROE*	5,704	0.063	0.237	–5.912	0.071	3.597
*LEV*	5,704	0.433	0.207	0.014	0.425	1.256
*GROW*	5,704	0.632	2.790	–9.951	0.190	86.720
*CRAA*	5,704	0.040	0.080	–1.938	0.040	0.661
*INV*	5,704	0.052	0.083	–0.406	0.031	2.384
*ADM*	5,704	0.097	0.332	–2.374	0.058	20.233
*DCOST*	5,704	94.290	180.273	1.000	60.000	4050.000
*IDRATIO*	5,704	0.376	0.056	0.200	0.364	0.800
*SHRCRL*	5,704	35.850	15.394	3.692	33.816	88.553
*INHOLDS*	5,704	4.300	4.731	0.000	3.050	59.480
*MSHARE*	5,704	0.066	0.131	0.000	0.001	0.735
*EPS*	5,704	0.371	0.536	–5.845	0.292	8.112
*BPS*	5,704	4.778	3.169	–0.918	4.097	45.073
*BTM*	5,704	0.466	0.239	0.013	0.430	1.403
*TRADE*	5,704	9.294	10.932	0.060	5.777	130.804

## Results

Table 3 shows the regression results of Models (1) and 2 that examine the effects of smog pollution on investor sentiment, and the moderating effects of media tone. The regression results of Model (1) show that the coefficient of *PM*_2.5_ is -8.9972 10^–3^, which is significant at the 5% level and indicates a negative impact of smog pollution on investor sentiment. In Model (2), the coefficient of *TONES* is calculated as 1.703×10^––51^, which is significant at the 1% level, indicating that a more positive media tone can create a more positive investor sentiment. The regression results of the interaction of *PM*_2.5_ and *TONES* in Model (2) show that the coefficient of *PM*_2.5_×*TONES* is 0.287×10^–4^, which is significant at the 5% level. Because the coefficient of *PM*_2.5_ in Model (1) is significantly negative and the coefficient of *PM*_2.5_×*TONES* in Model (2) is significantly positive, it can be concluded that media tone inhibits the negative influence of smog pollution on investor sentiment. Therefore, Hypotheses 1 and 2 are supported.

**TABLE 3:** The impact of PM2.5 on investor sentiment and moderating effect of media tone.

Variable	Model (1)	Model (2)
	*IS*	*IS*
*PM*_2.5_ (10^–4^)	−89.972**	−96.691**
	(−2.18)	(−2.28)
*TONES* (10^–4^)		−14.814***
		(−2.65)
*PM*_2.5_*TONES* (10^–4^)		0.287**
		(2.53)
*SOE*	0.036	0.038
	(0.30)	(0.31)
*SIZE*	−1.139***	−1.133***
	(−5.21)	(−5.20)
*AGE*	0.295***	0.291***
	(8.16)	(7.96)
*ROE*	1.670	1.676
	(1.43)	(1.43)
*LEV*	−0.272	−0.274
	(−0.32)	(−0.32)
*GROW*	−0.020	−0.020
	(−0.85)	(−0.85)
*CRAA*	−0.280	−0.288
	(−0.22)	(−0.22)
*INV*	0.406	0.394
	(0.58)	(0.56)
*ADM*	0.177	0.176
	(0.77)	(0.77)
*DCOST* (10^–4^)	−1.476	−1.217
	(−0.50)	(−0.40)
*IDRATIO*	0.962	0.977
	(1.17)	(1.18)
*SHRCRL* (10^–4^)	−2.685	−2.866
	(−0.08)	(−0.09)
*INHOLDS*	0.021***	0.021***
	(2.74)	(2.72)
*MSHARE*	−0.805	−0.800
	(−1.63)	(−1.62)
*EPS*	0.112	0.111
	(1.13)	(1.11)
*BPS*	−0.018	−0.019
	(−0.80)	(−0.84)
*BTM*	−4.485***	−4.479***
	(−10.05)	(−9.99)
*TRADE*	0.052***	0.053***
	(8.79)	(8.79)
Constant	21.970***	21.945***
	(5.14)	(5.08)
Control year	Yes	Yes
Control industry	Yes	Yes
N	5,704	5,704
Adj-*R*^2^	0.281	0.283
*F*	35.24	33.21

***, **, and * represent statistically significant at the 1, 5, and 10% levels respectively; brackets are t-values. In order to better reflect the results, the effective number of bits has been adjusted.

Empirical results indicate that investors have certain negative expectations for listed companies and underestimate the value of listed company located in smog- polluted areas. However, a positive tone from media can inhibit the negative investor expectations caused by smog pollution. These results are consistent with the literature on smog pollution in terms of firm value loss (Li et al., 2018) from negative influences on earnings as well as form decreased information content of company earnings in market valuations (Peng et al., 2021).

## Discussion

Various methods were used to ensure robustness in the conclusions of this study.

### Alternative Proxy Variable for Smog Pollution

To prove that the selection of the independent variable index does not affect the research conclusion, we apply PM_10_ to replace PM_2.5_ as the alternative proxy variable for smog pollution to test Hypothesis 1 and Hypothesis 2. Resulting estimates show the robustness of Hypotheses 1 and 2 is supported.

### Alternative Proxy Variable for Investor Sentiment

To prove that the selection of the dependent variable index does not affect the research conclusion, we apply the method of Chan et al. (2006) to replace Polk and Sapienza (2009) of calculating the discretionary component of discretionary accruals as the alternative proxy variable for investor sentiment. Resulting estimates show that Hypotheses 1 and 2 hold.

### Alternative Proxy Variable for Media Tone

To prove that the selection of the moderating variable index does not affect the research conclusion, we apply a weighted adjustment of the original probability of the overall sentiment orientation of the news as the alternative proxy variable for media tone. The estimation results indicate that the two hypotheses in this study are robust.

## Conclusion

This study on the psychological impact of smog pollution on investors finds that smog pollution in a company’s location can negatively affect investor sentiment, and that the tone of related news reportage can weaken this negative impact. This study highlights the effects of changes in the external natural environment on investor sentiment and is significant in maintaining the sustainable development of financial markets.

The findings point toward the following policy implications: (1) As the main governing body in smog pollution regulation and as the micro-subjects affected by smog pollution, companies should take the initiative in bearing the social responsibilities of pollution prevention and ecological protection. (2) Investors should take a rational, guiding role in the tone of related news reporting, and (3) regulatory authorities should guard against the expansion of the negative tone in news reports.

## Data Availability Statement

The original contributions presented in the study are included in the article/supplementary material, further inquiries can be directed to the corresponding author/s.

## Ethics Statement

This study was conducted in accordance with the recommendations of the Ethics Committee of the Beijing University of Chemical Technology.

## Author Contributions

BL and YW performed the initial analyses and wrote the manuscript. YL and TZ assisted in the data collection, data analysis, and textual modifications. All authors revised and approved the submitted version of the manuscript.

## Conflict of Interest

The authors declare that the research was conducted in the absence of any commercial or financial relationships that could be construed as a potential conflict of interest.

## Publisher’s Note

All claims expressed in this article are solely those of the authors and do not necessarily represent those of their affiliated organizations, or those of the publisher, the editors and the reviewers. Any product that may be evaluated in this article, or claim that may be made by its manufacturer, is not guaranteed or endorsed by the publisher.

## Footnotes

1 It is calculated according to the coefficient of *TONES* and *PM*_2.5_×*TONES* and the average value of *PM*_2.5_, the same as below.
